# Effectiveness of a Self-Monitoring Quality of Life Intervention For Patients with Cancer Receiving Palliative Care: A Randomized Controlled Clinical Trial

**DOI:** 10.31557/APJCP.2019.20.9.2795

**Published:** 2019

**Authors:** Ayako Matsuda, Yosuke Yamada, Noriko Ishizuka, Eisuke Matsushima, Kunihiko Kobayashi, Takayoshi Ohkubo, Kazue Yamaoka

**Affiliations:** 1 *Teikyo University School of Medicine, Department of Hygiene and Public Health,*; 2 *Toshima Hospital, Division Chief of Palliative Care Unit,*; 3 *Tokyo Medical and Dental University, Graduate School of Medical and Dental Sciences, Section of Liaison Psychiatry and Palliative Medicine, *; 4 *Teikyo University Graduate School of Public Health, Tokyo,*; 5 *Saitama Medical University International Medical Center, Department of General Thoracic Surgery, Saitama, Japan.*

**Keywords:** Cancer, quality of life, palliative care, self-monitoring

## Abstract

**Background:**

Use of patient-reported outcome measures in routine clinical practice has important benefits for patients with cancer. To examine the effect of a self-monitoring quality of life (QOL) intervention on global QOL and physical and emotional function in patients with cancer receiving palliative care.

**Methods:**

Prospective randomized study had been undertaken at Toshima Hospital, Japan. This study compared an intervention group that completed the shortened Care Notebook booklet versus a control group that received usual care. The primary outcome was global QOL and secondary outcomes were physical and emotional function. Participants completed the European Organization for Research and Treatment of Cancer Quality of Life Questionnaire Core 15 Palliative at baseline, and at 1 and 3 weeks. The effects of the intervention were evaluated with a linear mixed-effects model.

**Results:**

Forty-three patients were randomized. One patient in each group could not receive the allocated intervention, leaving 41 patients for inclusion in the modified intention-to-treat (ITT) analysis for the primary outcome. Twenty-seven patients were analyzed for the secondary outcomes using per protocol set (PPS). The ITT analysis showed no significant overall effect on global QOL (P=0.285), but the PPS analysis showed a significant overall effect on global QOL (P=0.034) and physical function (P=0.047) for group difference over time in the linear mixed-effects model.

**Conclusions:**

Use of the Care Notebook might have beneficial effects. The results could be interpreted as the effectiveness of the intervention of the Care Notebook for with cancer receiving palliative care.

## Introduction

Subjective assessments, including quality of life (QOL), are as important for patients with cancer as objective assessments (e.g., survival and response rates) (Matsuda et al., 2014). QOL assessment is often used as an outcome measure in clinical trials. A future task in this context is how QOL assessment information is fed back to patients. Cancer diagnosis and treatment are known to affect patients’ QOL (Hollen and Gralla, 1996; Mystakidou et al., 2005). However, medical staff may not sufficiently understand patients’ problems and difficulties, including the effect on their QOL. Previous studies suggested that use of patient-reported outcome measures in routine clinical practice has important benefits for patients with cancer, and feedback regarding QOL information improves doctor-patient communication and clinical decision making (Rubenstein et al., 1995; Espallargues et al., 2000; Detmar et al., 2002; Gilbody et al., 2002; Velikova et al., 2004; Greenhalgh et al., 2005; Boyes et al., 2006; Valderas et al., 2008; Fischer et al., 2012). However, these effects have not been clarified among patients with cancer who are receiving palliative care.

To measure patients’ QOL in daily clinical oncology practice, Kobayashi and colleagues developed the Care Notebook, and examined its validity and reliability (Kobayashi et al., 2005). The Care Notebook allows clinical oncologists to easily and repeatedly collect QOL information on physical function, mental function, and overall life wellbeing with minimal patient burden (Kobayashi et al., 2005). The Medical Oncology Department of Leiden University Medical Centre in the Netherlands developed a self-monitored QOL intervention for routine clinical practice based on the Care Notebook. However, use of the Care Notebook in clinical practice has not yet been examined among patients receiving palliative care. Based on a survey by the Japan Society of Gynecologic Palliative Medicine, Futagami et al., (2016) reported that regional alliance systems providing end-of-life care for patients with incurable gynecologic cancer are not sufficiently established in Japan. 

There is little research available worldwide on QOL interventions for patients with cancer in palliative care, and our study may help to address this lack. The primary objective of this study was to examine the effects of the Care Notebook as a routine self-monitoring QOL intervention to improve patient-reported global QOL in patients with cancer receiving palliative care. Secondary objectives were to examine the effects of the Care Notebook on improvement of patient-reported physical and emotional function.

## Materials and Methods

Further details of this study (e.g., recruitment procedure, sample size calculations, data management, conditions for discontinuation and actions in that event, monitoring, protocol amendments, ethics, and dissemination) are included in our published protocol (Matsuda et al., 2018). 

*Study design*

**Figure 1:**
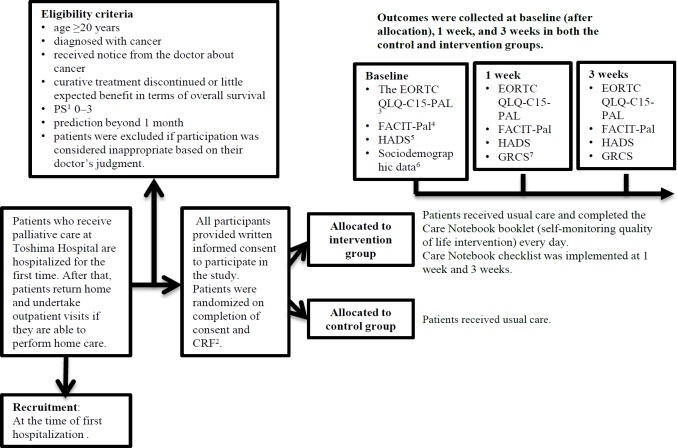
The Study Flow. ^1^PS, performance status; ^2^CRF, case report form; ^3^EORTC QLQ-C15-PAL: European Organization for Research and Treatment of Cancer Quality of Life Questionnaire Core 15 Palliative; ^4^FACIT-Pal, Functional Assessment of Chronic Illness Therapy-Palliative Care; ^5^HADS, Hospital Anxiety and Depression Scale; ^6^Sociodemographic data, sex, age, occupation, education, marital status, and children (yes/no); ^7^ GRCS: Global Rating of Change Scale

The design of this trial followed the Standard Protocol Items: Recommendations for Interventional Trials (SPIRIT) 2013 statement (Chan et al., 2013). This prospective randomized study was conducted at Toshima Hospital from May 2015 to December 2018. All participants were asked to provide written informed consent to participate in this study. After completion of consent and a case report form (CRF) by the present researchers, participating patients were randomized to an intervention group or a control group (usual care) (Figure 1). 

The CRF covered items such as date of birth, age, sex, diagnosis, Eastern Cooperative Oncology Group performance status (PS), first hospitalized day, cancer stage, previous history, complications, end day of aggressive therapy, treatment details (surgery [yes/no], radiotherapy [yes/no], chemotherapy [yes/no]), and date of death.

*Study setting*

This trial was approved by Toshima Hospital (No 26-11) and the Tokyo Medical and Dental University Ethics Committee (No 1756). After obtaining approval, the trial was registered with the UMIN clinical trials registry (Trial registration number: UMIN000025322. Issue date: May 31, 2017). Patients who receive palliative care at Toshima Hospital are first hospitalized. If they are able to perform home care, they return home and visit the hospital as outpatients. Recruitment was set at the time of first hospitalization.

*Participants and eligibility criteria*

Eligibility criteria included patients aged 20 years or older who were in a physical condition that allowed them to tolerate the investigation, were diagnosed with cancer and had received a notice about cancer from their doctor, with discontinued curative treatment or little expected benefit in terms of overall survival (PS 0–3), and had prediction of survival beyond 1 month.

*Randomization and blinding*

Eligible participants were randomly assigned to the intervention or control group using a permuted-block technique with a randomization list (random permutated blocks with a block size of four) (Stephen et al., 2001). Allocation to the intervention group was performed by the principal researcher. Eligible participants and researchers were not informed to which group participants were randomized, but blinding was not possible.

*Intervention*

*Intervention group*

Patients randomly assigned to the intervention group were asked to complete the Care Notebook booklet once each day in addition to usual care. The overall aim of the intervention was to inform a communication system between patients and medical staff. To promote communication, medical staff needs to understand the patient’s status and patients need to understand their condition, including QOL. We used the Care Notebook to achieve this. The Care Notebook (Care Notebook Center: http://www.care-notebook.com/en/download.html) was designed to assess QOL among patients with cancer, and has been validated and reported. We used the shortened Care Notebook questionnaire (hereinafter referred to as “Care Notebook”) for patients with cancer in palliative care. The Care Notebook is a self-administered, cancer-specific questionnaire that asks about patients’ conditions using 14 items structured in multidimensional scales. The questionnaire comprises three major scales: physical wellbeing, mental wellbeing, and life wellbeing. These scales are divided into several subscales. For example, physical wellbeing has three multi-item subscales (appetite loss, constipation, and fatigue), three single-item measures (pain, shortness of breath, and sleeping trouble), and also measures subjective QOL. The Care Notebook for the three weeks was bundled in a booklet form. In this study, the Care Notebook was used as a self-monitoring QOL intervention for routine clinical practice. The Care Notebook booklet could also be used as a diary to support communication between patients and medical staff.

**Table 1:** Baseline Characteristics in the Modified Intention to Treat Population

	Intervention group (N=20)		Control group (N=21)	
Age, mean(SD)	68.2	(11.7)	72.4	(10.6)
Sex, n(%)				
Men	10	(50.0)	7	(33.3)
Women	10	(50.0)	14	(66.7)
Cancer, n(%)				
Lung	3	(15.0)	6	(28.6)
Gastric	0	(0.0)	3	(14.3)
Ovarian	3	(15.0)	2	(9.5)
Pancreatic	1	(5.0)	2	(9.5)
Breast	1	(5.0)	2	(9.5)
Other	12	(60.0)	6	(28.6)
Receiving radiotherapy, n(%)				
Yes	7	(35.0)	7	(33.3)
No	9	(45.0)	9	(42.9)
Missing	4	(20.0)	5	(23.8)
Receiving chemotherapy, n(%)				
Yes	17	(85.0)	15	(71.4)
No	1	(5.0)	1	(4.8)
Missing	2	(10.0)	5	(23.8)
Eastern Co-operative Oncology Group performance status, n(%)				
0	2	(10.0)	1	(4.8)
1	2	(10.0)	5	(23.8)
2	9	(45.0)	7	(33.3)
3	6	(30.0)	6	(28.6)
Missing	2	(10.0)	2	(9.5)
Employment, n(%)				
Job at present	1	(5.0)	3	(14.3)
No	16	(80.0)	13	(61.9)
Other	3	(15.0)	4	(19.0)
Missing	0	(0.0)	1	(4.8)
Marital status, n(%)				
Married	11	(55.0)	14	(66.7)
Widowed	4	(20.0)	2	(9.5)
Divorced	2	(10.0)	0	(0.0)
Single	3	(15.0)	4	(19.0)
Missing	0	(0.0)	1	(4.8)
Baseline score of EORTC QLQ-C-15 PAL				
Global QOL, mean(SD)	41.7	(24.5)	43.7	(23.3)

SD, standard deviation; EORTC QLQ-C-15 PAL, European Organization for Research and Treatment of Cancer Quality of Life Questionnaire Core 15 Palliative

**Table 2:** Primary Outcome: Linear Mixed-Effects Model for Global Quality of Life Scores Over Time

Global QOL	Estimate of Effects	(SE)	95%CI	P value
modified ITT				
Intercept	49.82	(27.41)	-5.74 to 105.39	0.077
Group a	-8.02	(9.01)	-26.15 to 10.10	0.378
Global QOL score at baseline	-0.75	(0.19)	-1.14 to -0.37	<0.001
Age	-0.03	(0.40)	-0.85 to 0.78	0.933
Sex ^b^	8.60	(8.75)	-9.14 to 26.3	0.332
Time ^c^	-2.39	(4.76)	-12.01 to 7.23	0.618
Group×time	7.39	(6.81)	-6.39 to 21.17	0.285
PPS				
Intercept	44.30	(25.45)	-7.89 to 96.48	0.093
Group a	-14.43	(8.83)	-32.23 to 3.41	0.110
Global QOL score at baseline	-1.13	(0.21)	-1.55 to -0.71	<0.001
Age	0.29	(0.40)	-0.52 to 1.10	0.466
Sex ^b^	5.14	(8.24)	-11.75 to 22.04	0.537
Time ^c^	-2.18	(6.35)	-15.22 to 10.86	0.734
Group×time	20.41	(9.15)	1.63 to 39.19	0.034

ITT, intention to treat; PPS, per protocol set; SE, standard error; QOL, quality of life; CI, confidence interval; ^a^ Group, Intervention=1; control=0; ^b ^Sex, Men=1; Women=0; ^c^ Time: 1 week=2; 3 week=3

**Table 3:** Secondary Outcomes: Linear Mixed-Effects Model for Physical and Emotional Function Scores Over Time

	Estimate of Effects	(SE)	95%CI	P value
Physical function				
Intercept	15.90	(22.83)	-30.94 to 62.73	0.492
Group a	1.24	(8.33)	-15.51 to 17.99	0.882
Global QOL score at baseline	-0.48	(0.11)	-0.71 to -0.25	<0.001
Age	0.03	(0.31)	-0.60 to 0.67	0.913
Sex ^b^	10.35	(6.92)	-3.86 to 24.56	0.147
Time ^c^	-7.21	(6.88)	-21.33 to 6.90	0.304
Group×time	20.53	(9.86)	0.31 to 40.74	0.047
Emotional function				
Intercept	59.10	(17.61)	21.54 to 96.67	0.004
Group a	-11.55	(6.98)	-25.58 to 2.48	0.105
Global QOL score at baseline	-0.93	(0.11)	-1.17 to -0.68	<0.001
Age	0.20	(0.40)	-0.52 to 1.10	0.466
Sex ^b^	5.14	(0.27)	-0.38 to 0.79	0.514
Time ^c^	1.89	(6.92)	-12.68 to 16.46	0.788
Group×time	6.35	(9.71)	-14.16 to 26.86	0.522

Secondary outcomes analyzed following the per protocol set principle; QOL, quality of life; SE, standard error; CI, confidence interval; ^a^Group, intervention=1; control=0; ^b^ Sex, men=1; women=0; ^c ^Time, 1 week=2; 3 week=3

**Figure 2:**
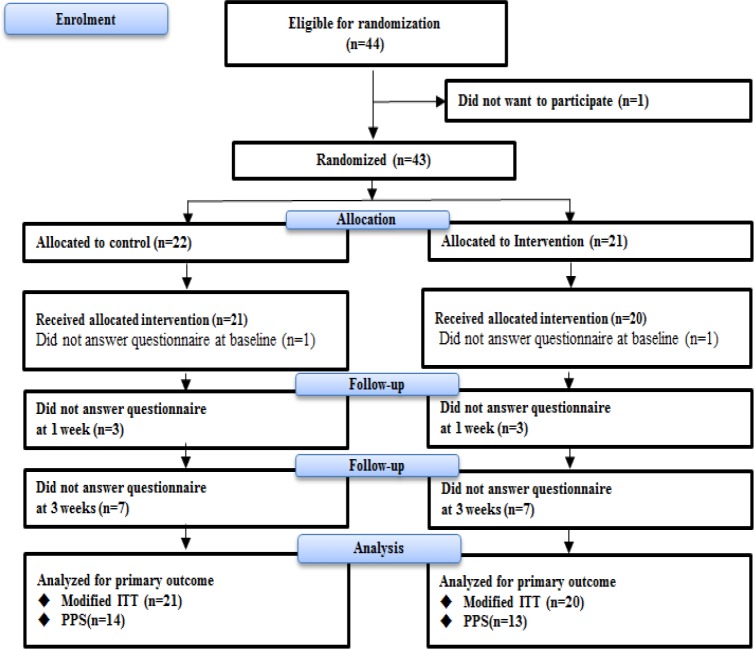
Flow through the Study. ITT, intention to treat; PPS, per protocol set

**Figure 3:**
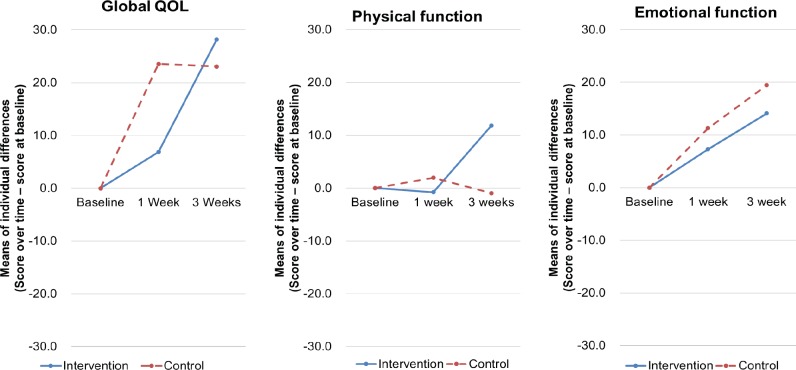
Changes of European Organization for Research and Treatment of Cancer Quality of Life Questionnaire Core 15 Palliative Scores of Patients Over Time. Mean values of individual changes in per protocol set analysis

*Control group*

The control group received “usual care” or routine practice that medical staff provided during course of routine clinical care for patients with cancer receiving palliative care.

*Study hypothesis*

The hypothesis underlying this study was that patients with cancer who were receiving palliative care and completed the Care Notebook booklet (intervention group) would have a better global QOL than those who received only usual care (control group). 

*Outcome measures*

*Primary outcome*

The primary outcome was global health status/QOL (global QOL) as reported by patients, assessed by using the European Organization for Research and Treatment of Cancer Quality of Life Questionnaire Core 15 Palliative (EORTC QLQ-C-15-PAL) (Groenvold et al., 2006; Groenvold et al., 2006; Arraras et al., 2014; Echteld et al., 2006). A global QOL score of 100 indicates the best possible QOL. 

*Secondary outcomes*

Secondary outcomes were physical and emotional function, as measured by the EORTC QLQ-C15-PAL. A score of 100 in both physical and mental wellbeing indicates the best possible function or QOL. 

*Data collection and time points*

Participants’ sociodemographic data were collected at baseline (after allocation). Data for the primary and secondary outcomes were collected at baseline (after allocation), at 1 week, and at 3 weeks in both the control and intervention groups (Figure 1).

*Participant characteristics*

Sociodemographic data including sex, age, employment, and marital status were obtained from the CRF. Clinical data, including diagnostic names, PS, treatment details (radiotherapy, chemotherapy) were also obtained from the CRF.

*Statistical analysis*

The primary outcome was analyzed following the modified intention to treat (ITT) principle, with per protocol set (PPS) performed as sensitivity analyses. For the modified ITT (Dossing et al., 2016), we excluded patients who did not answer questionnaire at baseline after the allocation. A previous study showed that the palliative-modified ITT analysis allowed a systematically less biased approach to evaluating the effects of interventions being evaluated in Phase III hospice/palliative care trials (Currow et al., 2012). The last observation carried forward method was used to manage missing data due to attrition. For the PPS, we excluded patients with poor compliance to the study protocol or who refused to receive the allocated Care Notebook booklet after baseline. The secondary outcomes were analyzed following the PPS principle.

A linear mixed-effects model was used to compare the effects of the intervention on the primary and secondary outcomes (Brow and Prescott, 1999). The model included the changes in global QOL from baseline as the outcome variable; “*baseline score for global QOL*” as a covariate; “*age*”, “*sex*”, “*time*,” and “*group*,” as fixed effects; “*patient*” as random effects; and “*Group×time*” as an interaction term. Time was fitted into the model as a categorical variable. Similar models were fitted with the changes in scores for physical or emotional function from baseline to 3 weeks. P < 0.05 (two-sided) was considered statistically significant. Statistical analyses were performed with SAS version 9.4.

## Results

*Patients*

The study protocol for our study has previously been published (Matsuda et al., 2018). However, the study was terminated because of a change in the establishment of the research framework for resignation of main researcher for health reasons. Therefore, we were unable to recruit the final participants. The present article reports the results of an analysis involving 44 participants who we were able to recruit.

Patients were included from May 2015 to December 2018. A flow-chart of randomized participating patients is presented in Figure 2. Forty-four patients were considered eligible, and 43 patients were randomized. Twenty-two patients were allocated to the control group and 21 patients to the intervention group. One patient in each group could not answer the questionnaires at baseline after group allocation, leaving 41 patients for inclusion in the analysis for the primary outcome. In addition, 35 patients answered the follow-up questionnaire at 1-week and 27 answered the questionnaire at 3-weeks. Therefore, 27 patients were analyzed for the secondary outcomes. The characteristics of participating patients at baseline are shown in Table 1. The mean age in the intervention group was 68.2 years and in the control group was 72.4 years. About half were females and half were married. Lung cancer was the most common cancer type and the majority of patients were receiving chemotherapy. The mean global QOL score (standard deviation) in the EORTC QLQ-C-15-PAL at baseline for patients in the intervention and control groups was 41.7 (24.5) and 43.7 (23.3), respectively. There was no statistically significant differences in the baseline characteristics between the intervention group and control group.

*Primary and secondary outcomes*

The results of the linear mixed-effects model for the changes in global QOL from baseline are presented in Table 2 (modified ITT and PPS). Figure 3 illustrates the changes from baseline in global QOL and physical and emotional function over time for the PPS analyses. The modified ITT analysis showed no significant overall effect on global QOL for group difference over time in the linear mixed-effects model (P=0.285). However, the PPS analysis showed a significant overall effect on global QOL for group difference over time in the linear mixed-effects model (P=0.034). The intervention group showed better scores than the control group at 3 weeks (Figure 3). The results of the linear mixed-effects model for the changes from baseline in physical and emotional function are presented in Table 3 (PPS). A significant overall effect on physical function was observed for group difference over time in the linear mixed-effects model (P=0.047), but no significant overall effect on emotional function was observed for group difference over time (P=0.522). The intervention group showed better scores for physical function than the control group at 3 weeks. Increasing trends for the intervention group and the control group were observed for emotional function (Figure 3).

## Discussion

*Main findings*

The PPS analysis in our study showed that routine use of the Care Notebook as a self-monitoring QOL intervention for patients with cancer receiving palliative care improved patients’ global QOL and physical function scores (as assessed by the EORTC QLQ-C-15-PAL).

*Strengths and limitations of this study*

A strength of our study lies in its contribution to the body of knowledge of effective, patient-reported QOL measures for patients with cancer receiving palliative care. There is a paucity of research in this area, and this is the first study to investigate the use of the Care Notebook among patients with cancer receiving palliative care. This study also had some limitations. The main limitation was that we were unable to recruit the planned number of patients. In our published study protocol, we intended to use a two-sided significance level of 5% and a power of 90%, assuming a dropout rate of 30% (effect size 0.56) (Matsuda et al., 2018). In this study, the power was 70% (effect size 0.99; intervention group: mean change for global QOL score from baseline [month 1−baseline] 28.2, standard deviation 28.4). In addition, we excluded patients with poor compliance to the study protocol. We considered that our PPS analysis showed significant overall positive effects on global QOL because the effect size was higher than we expected in our published study protocol. Another limitation was that this study did not use a double-blind design. However, eligible patients and researchers were not informed of the group to which patients were randomized until the study started. In addition, the outcome measures were clearly set, this study used reliable scales, and the test had adequate power to detect the effect of the intervention on the outcomes.

*What this study adds*

Our results regarding the mean score for global QOL at baseline were consistent with the global QOL score (mean 41.4, standard deviation 24.6) in a multicenter observational study conducted by the Japanese Organization for Research and Treatment of Cancer (JORTC PAL-09) (Iwase et al., 2015). 

In our study, use of the Care Notebook as a self-monitoring QOL intervention in routine clinical practice had a positive effect on participating patients’ global QOL and physical function (PPS analysis). Our findings help to address the gap in research available worldwide on QOL interventions for patients with cancer in palliative care. We clarified that use of the Care Notebook was effective and practical in this patient population. The small number of patients included in the present analysis may explain why our ITT analysis did not show beneficial effects of the self-monitoring QOL intervention using the Care Notebook. However, the beneficial effects of the intervention were shown in the PPS analyses for global QOL and physical function. About 65% of participating patients in this study could complete follow-up at 1- and 3-weeks, which suggests that the self-monitoring QOL intervention using the Care Notebook may be effective for patients who could complete the task for ≥3 weeks. A previous randomized controlled trial involving an intervention in early referral to a specialist palliative care team did not detect beneficial effects for patients with cancer in palliative care (Groenvold et al., 2017). In our study, the intervention covered both the early stage and during the illness period. Therefore, we suggest that patients with cancer in palliative care may need to receive a continuous intervention from an early stage. Erharter et al., (2017) reported that computer-based QOL monitoring was useful for early detection of physical symptoms and psychosocial problems. In our research, the Care Notebook was used as a daily clinical routine in the palliative care program. Our findings suggest that medical staff may use the Care Notebook as a routine self-monitoring QOL intervention in clinical practice for patients with cancer receiving palliative care. A previous study also showed that routine assessment of health-related QOL among patients with cancer had an impact on physician-patient communication and resulted in benefits for some patients who had better health-related QOL (Velikova et al., 2004; Epstein et al., 2017). Routine use of the Care Notebook in clinical practice may also facilitate such improvements in physician-patient communication in the palliative care setting. Further studies will be necessary to investigate the use of a routine self-monitoring intervention based on the Care Notebook among patients with cancer receiving palliative care in different countries and contexts. Further studies should also consider the practical use of the Care Notebook in this patient population.

Our study might clarify the practical use of the Care Notebook for patients with cancer receiving palliative care. This self-monitoring QOL intervention showed improvement in global QOL and physical function among patients in this population. Worldwide, there are limited QOL interventions available for patients with cancer in palliative care, and the Care Notebook could help to address this lack. We recommend use of the Care Notebook intervention for patients with cancer in healthcare facilities for palliative care. 

## Data sharing statement

The informed consent documents are available on request.
